# Antiphospholipid syndrome presenting as extensive skin ulcers on unilateral lower extremity: a case report

**DOI:** 10.3389/fsurg.2024.1360928

**Published:** 2024-04-09

**Authors:** Yanan Yang, Yaoyao Zhou, Lingyi Zhang, Mengru Pang

**Affiliations:** Department of Burns and Plastic Surgery, The Affiliated Hospital of Guizhou Medical University, Guiyang, China

**Keywords:** ulcers, lower extremity, antiphospholipid syndrome, surgery, immunity

## Abstract

Antiphospholipid syndrome (APS) is an autoimmune disorder characterized by recurrent arterial and venous thrombosis, habitual fetal miscarriages, often accompanied by mild to moderate thrombocytopenia, and persistent moderate-to-high titer positivity for antiphospholipid antibodies (aPLs). However, patients with antiphospholipid antibodies may also present with several nonthrombotic clinical manifestations, such as thrombocytopenia, cardiac valve disease, nephropathy, skin ulcers, or cognitive dysfunction, which are collectively referred to as nonstandard manifestations of APS. Of these, for APS with predominantly cutaneous ulcers, previous reports have focused on APS with combined cutaneous vasculitis, and its medical treatment, rather than cutaneous ulcers with predominantly fatty inflammatory lesions, and the associated surgical treatment. Here, we admitted a relatively rare case of primary APS with extensive skin ulceration of the right lower extremity, without cutaneous vasculitis, in the presence of extensive and severe inflammatory lipoatrophy, carrying anti-β2-glycoprotein I and lupus anticoagulant, which is reported as follows, with a view to raising awareness of this disease.

## Case presentation

A 46-year-old woman with “widespread swelling and ulceration of the right lower extremity for two months”. Two months ago, the anterior side of the right lower limb had a large area of redness and swelling, obvious subcutaneous swelling to the skin breakthrough she was diagnosed with a right lower limb infection in the local hospital, treated by adequate drainage, anti-infection and regular dress change, the wound did not heal, and further expansion. Transferred to our hospital, continued anti-infection treatment, VSD negative pressure suction treatment for one week, granulation tissue was seen, skin grafting to repair the wound. One week later, most of the skin survived. Then every subsequent dressing change saw gradual atrophy of the healed skin flakes and oozing of large amounts of yellowish clear fluid from the wound. Two weeks after the operation, the erythema of the right lower limb further expanded to the medial-lateral and part of the posterior side. A small amount of lesion tissue was taken and sent for examination, and an inflammatory reaction of adipose tissue was considered, with no manifestation of cutaneous vasculitis.

## Diagnosis

Blood tests for leukocytes, neutrophils, lymphocytes, and septicemia were normal. Multiple bacterial and fungal cultures were free. Vascular ultrasound of both lower extremities showed deep vein thrombosis of the left calf. The patient had no diabetes or lower extremity vasculopathy, and no history of immune system disorders such as systemic lupus erythematosus or rheumatoid arthritis. On further questioning of the medical history, the patient lost several family members one after another two months ago, and the patient suffered from heart failure and chronic insomnia.

**Figure 1:**
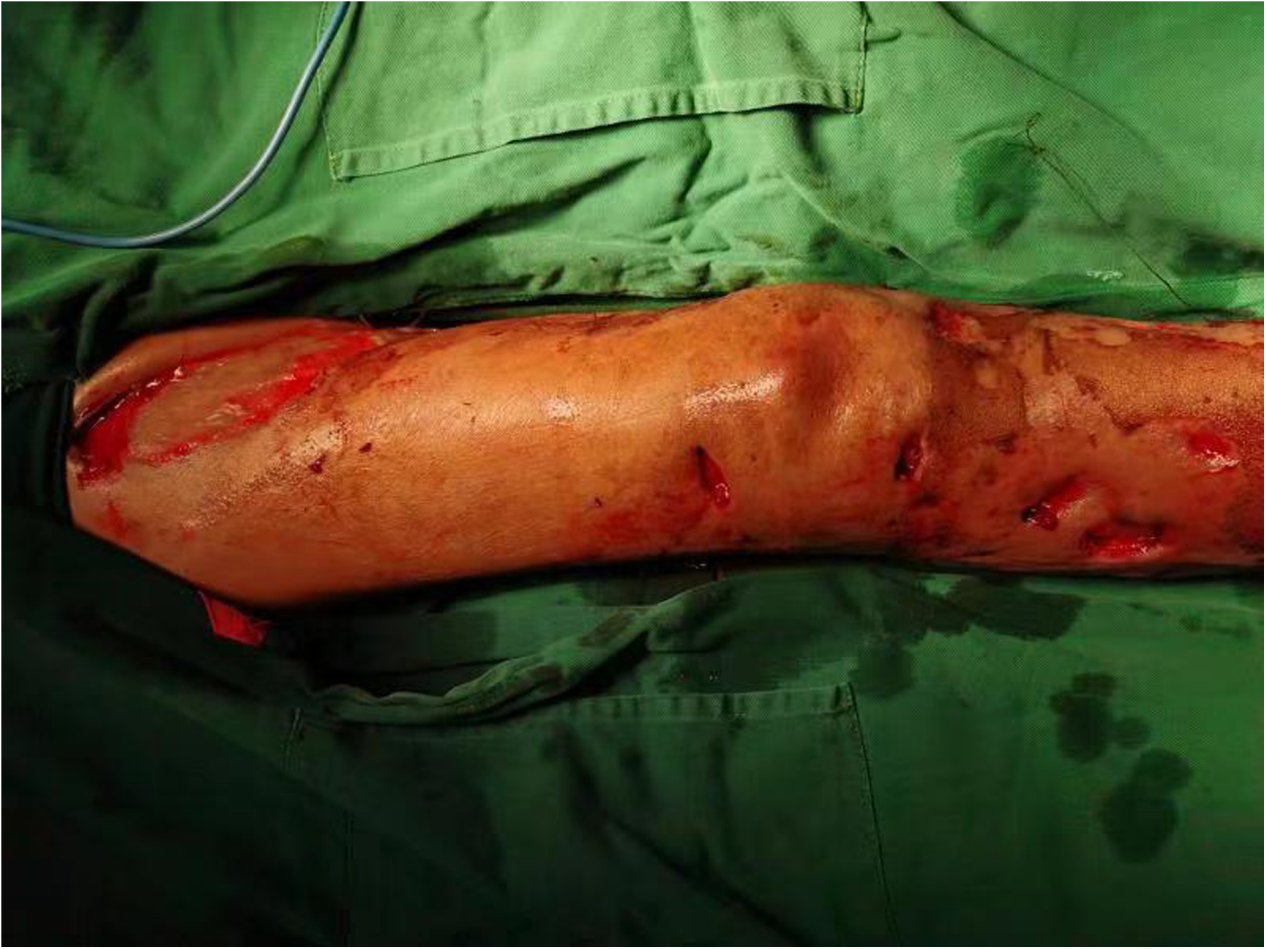
Extensive preoperative ulceration of the right lower extremity.

Combing the patient's symptoms, signs and experiences (family loss), Immunologists suggest that there may be immunodeficiency. It is recommended to check the ANA antibody profile, anti-neutrophil cytoplasmic antibodies, immunoglobulin + complement, rheumatoid factor, anti-cyclic citrullinated peptide antibodies, cardiolipin antibodies, and T-cell test for tuberculosis infection, which were all within normal limits. Multiple incisions and drains in the erythematous area of the right lower extremity exuded yellowish-clear fluid without purulent secretions (Figure 1). Immediately, the patient's whole body began to swell, he became febrile and had respiratory distress, and he was transferred to the ICU for continued treatment. The patient had a persistent high fever, around 40°C, and was comatose with ventilator-assisted respiration. The departments of infection, clinical pharmacy, and rheumatology and immunology were invited to consult and be recommended to intensify the anti-infective treatment and immune supportive treatment and to send the test of antiphospholipid antibody hexa test. The patient continued to have a high fever. A large amount of yellow-clear fluid is oozing from the incision and drainage of the right lower extremity. Subsequently, the results of 6 antiphospholipid antibodies were returned: anti-β2-glycoprotein I 70.40 ng/ml (normal range: 0.00–65.00) and lupus anticoagulation confirmation test 43.6 s (normal range: 30.0–38.0), and the rest were in the normal range. The Department of Rheumatology and Immunology made a final diagnosis of antiphospholipid syndrome according to the 2004 APS classification diagnostic criteria, based on pathological biopsy and symptoms and signs.

## Treatment

**Figure 2:**
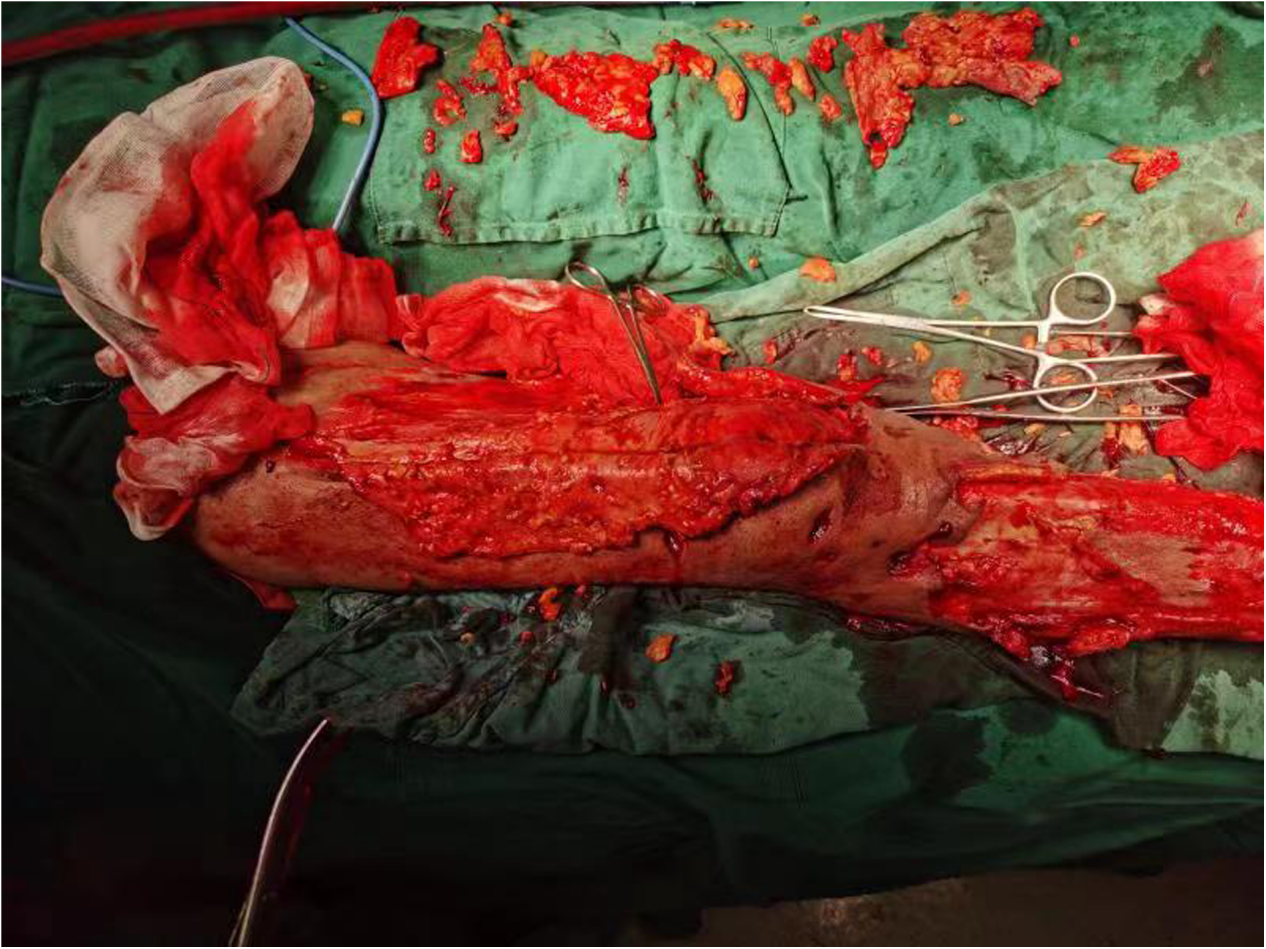
Extensive intraoperative removal of degenerated subcutaneous adipose tissue.

Before the diagnosis was definitive, the patient's general condition was critical and life-threatening, and the decision was made to perform emergency surgery to remove the inflamed tissue to control the symptoms. The patient's family agreed to the surgery immediately. Extensive subcutaneous adipose layer edema of the right lower limb was found, with a pale color and crunchy texture. Adipose tissue was removed and sent for pathological examination suggesting a severe inflammatory reaction: partial necrosis of the adipose lobules, a large number of histiocytes and multinucleated giant cell reaction, infiltration of a large number of lymphocytes and neutrophils, and formation of lipomatous membrane inflammation, with no vasculopathy or thrombosis seen. During surgery, the diseased adipose tissue was extensively and thoroughly removed (Figure 2), and then the medium-thick piece of skin was implanted into the wound. The temperature dropped to about 38°C and antibiotic use was lowered postoperative. Two days later, the temperature dropped to normal range. Then combined with the patient's clinical manifestations and laboratory tests, methylprednisolone (20 mg Qd) intravenous treatment, low molecular heparin calcium anticoagulation and gammaglobulin supportive therapy.

**Figure 3:**
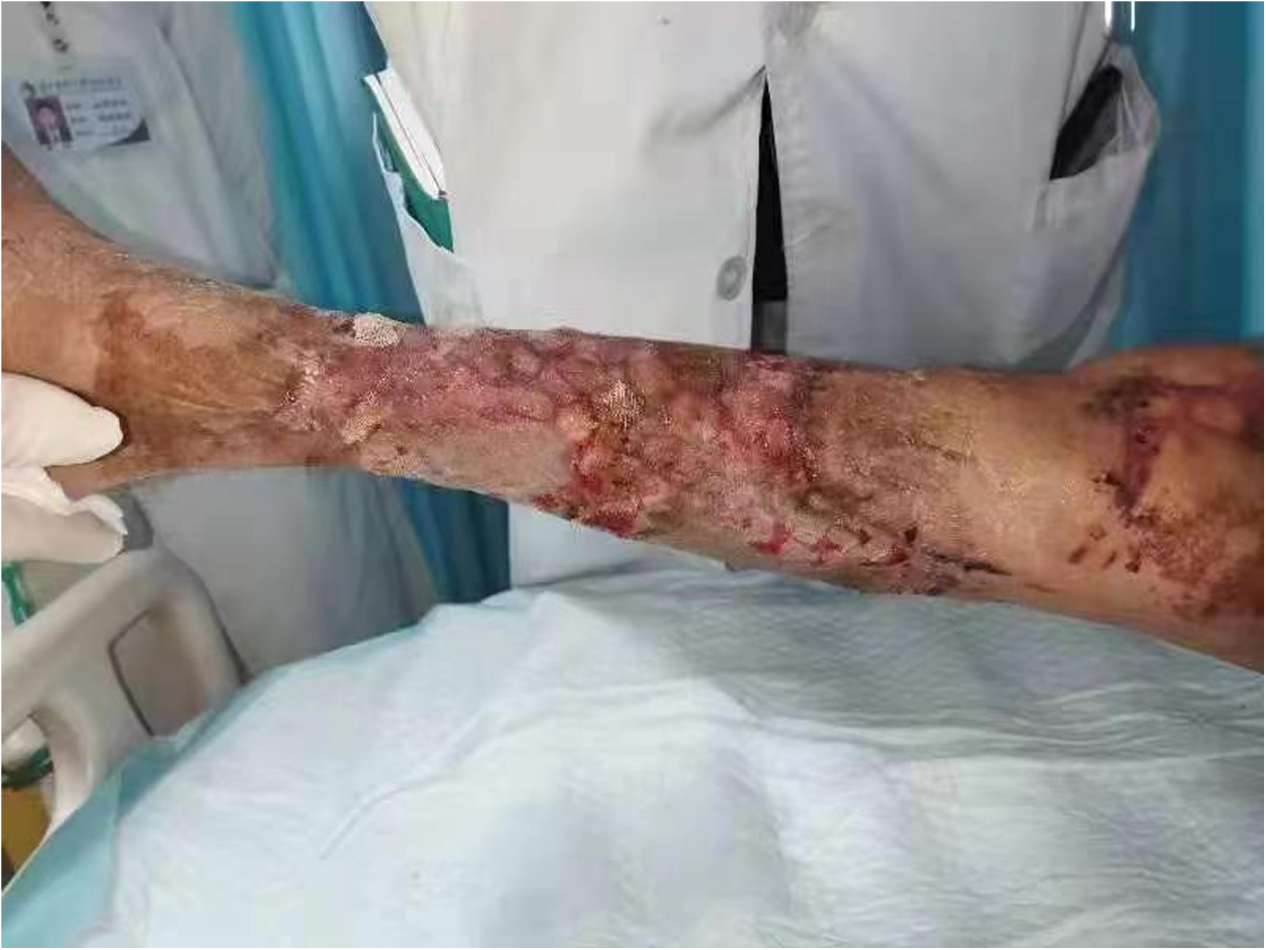
Postoperative right lower extremity ulcer wound repair 1 month after surgery.

One week later, the patient was off the ventilator, without dyspnea, and in better general condition (Figure 3). By monitoring the antiphospholipid antibodies, gradually reducing the dose of hormone use. Three months later, the patient's right lower limb ulcers had healed, and the ultrasound of the lower limb blood vessels showed that there was no deep vein thrombosis.

## Follow-up

**Figure 4:**
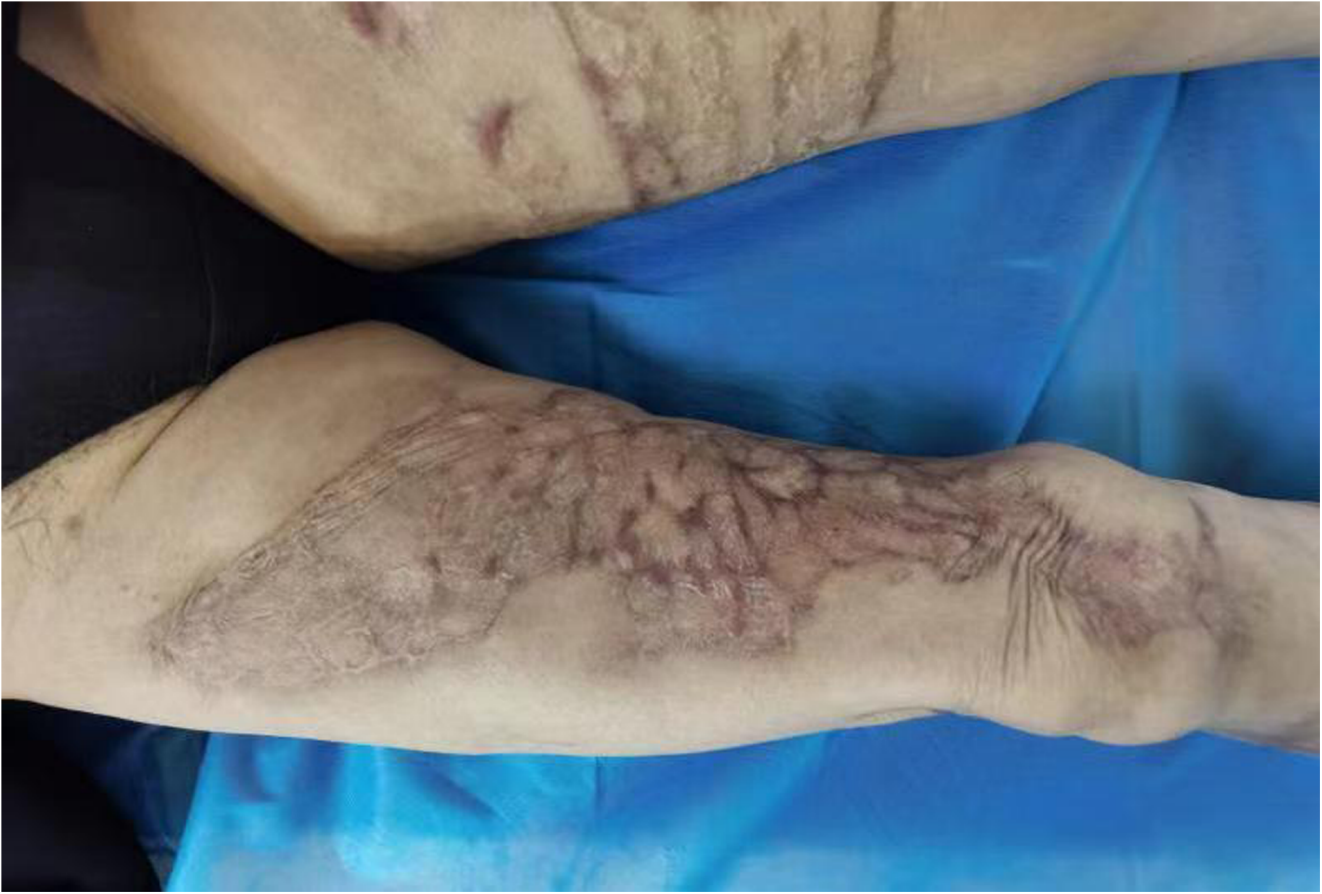
Status of the right lower extremity ulcer after healing one year after surgery.

Up to one year of follow-up, there was no recurrence of the right lower extremity ulcer (Figure 4), and the patient walked to our department with his family, and the patient indicated that there was no lower limb dysfunction except for obvious scarring and pigment changes (negative antiphospholipid antibody, oral methylprednisolone hydrochloroquine sulfate tablets with 4 mg Qd).

## Discussion

When unexplained lower extremity ulcers are present, vasculopathy, diabetes-related and tissue infectious lesions (necrotizing fasciitis) are generally considered clinically in the first instance, and after these causes have been ruled out, autoimmune abnormalities should be favored.

An antiphospholipid syndrome is characterized by persistently elevated antiphospholipid antibodies. Main clinical manifestations include Thrombocytopenia, Livedo reticularis, Stroke, Transient ischaemic attacks, Deep vein thrombosis, Pulmonary embolism, Epilepsy, Skin ulcers, Valve thickening/dysfunction, Vegetations, Myocardial infarction, Superficial thrombophlebitis and Autoimmune haemolytic anaemia, and the symptom of skin ulcer was reported occurs in about 1.7%–5.5%. However, there are no reports of massive necrosis of the skin, soft tissues and severe inflammatory reactions (1). This case reports a rare non-standard presentation of APS, the first clinical manifestation was only a large area of unilateral subcutaneous adipose tissue severe inflammation induced by extensive skin ulceration of the lower limbs, through the joint efforts of the Department of Rheumatology and Immunology, the Department of Critical Care Medicine and the Department of Plastic and Reconstructive Burn Surgery of the authors' department, to timely control the development of the patient's large area of necrosis of the lower limbs of the fat tissues induced by the APS and to clarify the cause of the disease, and stabilizing the vital signs while targeting treatment of the APS for three months after the development of the APS and the healing of the ulceration of the right lower limbs effectively control the development of the APS. Non-standard manifestations of APS have diverse clinical symptoms (2, 3), are difficult to diagnose, develop rapidly and even become life-threatening, and there is no uniform treatment protocol. This patient had experienced uncommon stressful events before the onset of the disease, which may be a trigger for the onset of autoimmune diseases (4). Acute stress may trigger a transient immunologic protective response, whereas the patient experienced the loss of several loved ones, and prolonged or poorly controlled sources of psychological stress may lead to diminished immune system function (5).

Therefore, understanding the patient's mental stress situation can also be a clue to consider autoimmune diseases in clinical practice. This case demonstrates the important role of surgical treatment in the treatment of severe APS, and it is important to remove the diseased tissue in time, stabilize the patient's vital signs, and gain a clear diagnosis and treatment time for the patient. At the same time, this case also demonstrates the importance of multidisciplinary team-integrated treatment, and strengthening the exchange and cooperation between various specialties is an inevitable trend to optimize the treatment in the future, especially for some atypical and dangerous diseases.

## Data Availability

The original contributions presented in the study are included in the article/Supplementary Material, further inquiries can be directed to the corresponding author.
